# Coping with Moral Dilemmas in German Criminal Law Theory and Justice: Classical Cases and Modern Variants

**DOI:** 10.1007/s10609-023-09452-0

**Published:** 2023-02-17

**Authors:** Frank Peter Schuster

**Affiliations:** Julius-Maximilians-Universität, Domerschulstr. 16, 97070 Würzburg, Germany

## Abstract

Dilemma scenarios have always been among the most common problems of moral philosophy and criminal law theory. One only has to contemplate the Plank of Carneades, the classic thought experiment whereby two shipwrecked people’s only hope of rescue is a floating board that can only be occupied by one person. Other scenarios are Welzel’s switchman case and the well-known Trolley Problem. In most of the debated cases the death of one or more people is absolutely unavoidable. The protagonists do not cause the situation but are fated to come into conflict. The focus of this article is on one recent and one future variant. First, the prioritization of medical aid (also known as “triage”) is the subject of intense debate, because the COVID-19 pandemic posed a permanent risk of a temporary collapse in the health system in several countries. Situations had arisen whereby some patients can no longer be treated owing to lack of capacity. It can be asked whether a decision to treat may be based on which patients have a better chance of survival, whether reckless previous behaviour may play a role, and whether a treatment, once started, may be discontinued in favour of another. Second, dilemma scenarios are also one of the last remaining (largely unresolved) legal difficulties of autonomous vehicles. Never before has a machine been given the power to determine the life or death of human beings. Even though the automotive industry promises that such situations will hardly ever occur, the problem could prove to be a tangible obstacle to acceptance and innovation. The article offers solutions for those distinct scenarios, but it is also intended to demonstrate the underlying legal concepts of German law: namely, the tripartite analysis of criminal law and the idea of human dignity as a fundamental principle of the German constitution.

## Introduction

Substantive criminal law in the legal system of common law still develops quite independently from that in civil law and vice versa.^1^ Nevertheless, quite often we deal with similar problems. For example, ethical dilemmas have always posed a special fascination for the public, as well as in moral philosophy and jurisprudence. There is much to suggest a role of the psychological phenomenon of “wanting to be scared”^2^ in this, and even philosophy professors and law teachers are not entirely immune. One only has to contemplate the *Plank of Carneades*, the well-known thought experiment whereby two shipwrecked people’s only hope of rescue is a floating board that can only be occupied by one individual.^3^ One castaway kills the other to secure the life-saving plank for himself and be rescued. The case may be found in every German textbook on criminal law.^4^ Real cases from the 19th century have been handed down from US-American^5^ and English^6^ maritime law. The English *Mignonette* case is a particularly well-known example in Germany.^7^ The case concerned cannibalism among shipwrecked people; the proceedings ended with the offenders being sentenced to death and later pardoned after serving six months in prison.^8^

However, there are much more recent scenarios. The pandemic triggered by the SARS-CoV-2 virus had led to temporary situations (e.g., in Italy and France), where hospital physicians had to decide which patients were allocated an intensive care bed with a ventilator (and thus the prospect of survival) and who had to die because they could no longer be treated owing to lack of capacity. The prioritization of medical assistance in emergency situations is known as “triage” (from the French word *trier* = to sort). Although the collapse of the German health care system has been averted, the vaccination campaign is showing clear successes and the current omicron variant seems to be quite harmless, the end of the pandemic is hardly foreseeable in view of possible new virus variants. In any case, coronavirus will not be the last pandemic or crisis in our lifetime. Resources are not unlimited, even in the Western world. So the problem of the allocation of intensive care resources is still the subject of intense debate.^9^ It can be asked whether the decision may be based on which patients have the better chance of survival, whether children and young people with greater life expectancy may be given preference over older people and those with previous illnesses, whether the vaccination status or previous reckless behaviour may influence the decision, and whether a treatment, once started, may be discontinued in favour of another.

In traffic, scenarios can also occur in which violations, human error, or natural phenomena lead to risky situations when accidents cannot be avoided. Highly and fully autonomous vehicles can record and process information very quickly. If an accident cannot be avoided, the computer is quite capable of considering all possible courses of action to minimize damage. Of course, the vehicle must always choose material damage over personal injury, but one can also ask whether the system may be programmed to keep the number of personal injuries as low as possible.^10^ At some point, the technical systems may be able to determine how many people are in a vehicle or even their age. However, the “right” decision cannot ultimately be determined by a machine or the automotive industry. To promote autonomous driving, the industry is rather dependent on acceptance by the population and a secure legal framework.

For some years now, a worldwide internet survey by US researchers – titled *Moral Machine* – has attracted attention. The results of a survey on autonomous driving were published in an article in *Nature*^11^ magazine. The vast majority of the test participants allowed the quantitative weighing of human life;^12^ the majority also showed a strong tendency to spare children over elderly people (although there are cultural differences in this respect)^13^ and judged whether the people concerned had obeyed the rules. There are also even more recent surveys concerning the allocation of ventilators to COVID-19 patients on a smaller basis, the results are similar, but not as clear.^14^

These two problems – the prioritization of medical aid (infra III.) and the emergency algorithms for autonomous vehicles (infra IV.) – are the focus of the following article.

## Legal Foundations and Classic Thought Experiments

### Legal Basics

The German criminal law – as well as many other legal systems that follow the same pattern^15^ – distinguishes between three levels of inquiry: satisfaction of all objective and subjective elements of the offense as defined in the statute (*Tatbestandsmäßigkeit*),^16^ the wrongfulness (*Rechtswidrigkeit*), and the culpability (*Schuld*) in the sense of “blameworthiness” of the actor.^17^ The three-step analysis of criminal liability may be familiar to some Anglo-American scholars, as it also appears in a few US textbooks.^18^

#### Statutory Rules Concerning Dilemma Scenarios

In consequence, the German Criminal Code (*Strafgesetzbuch* – *StGB*) contains (contrary to US-American § 3.02 Model Penal Code; English case law is also different^19^) two necessity regulations (choice of evils defences), to distinguish between justification (excluding wrongfulness) and excuse (excluding culpability).

a) Justifying Necessity (*Rechtfertigender Notstand*) is regulated in § 34 StGB; the provision reads as follows.

“Whoever, when faced with a present danger to life, limb, liberty, honor, property or another legal interest which cannot otherwise be averted, commits an act to avert the danger from themselves or another is not deemed to act unlawfully if, upon weighing the conflicting interests, in particular the affected legal interests and the degree of the danger facing them, the protected interest substantially outweighs the one interfered with. However, this only applies to the extent that the act committed is an adequate means to avert the danger.”

This general “lesser evil defence” was the last grounds of justification to be expressly integrated into written law and has been part of the German Criminal Code since 1973.^20^ However, after a decision by the Imperial Court of Justice (*Reichsgericht*) in 1927^21^ to legalize abortions performed to save the mother’s life or health, it has been recognized under customary law. Two further provisions from the Civil Code (*Bürgerliches Gesetzbuch* – *BGB*), namely §§ 228 and 904 BGB, are much older. They allow interference with the property of others but are not applicable to personal injury.^22^

b) Excusing Necessity (*Entschuldigender Notstand*) is regulated in § 35 StGB; the provision reads as follows.

“(1) Whoever, when faced with a present danger to life, limb or liberty which cannot otherwise be averted, commits an unlawful act to avert the danger from themselves, a relative or close person acts without guilt. This does not apply to the extent that the offender could be expected, under the circumstances, to accept the danger, in particular because said offender caused the danger or because of the existence of a special legal relationship; the penalty may, however, be mitigated pursuant to section 49 (1), unless the offender was required to accept the danger on account of the existence of a special legal relationship…”

The wording makes it clear that the provision only protects the perpetrator, if he tries to avert a danger to his own life, limb and personal liberty or that of a close person. However, “excusing necessity” may even be granted in cases where life stands against life.

#### Unwritten Legal Rules Concerning Dilemma Scenarios

In addition, in dilemmatic situations, two other institutes have to be taken into account; even so, the written law does not explicitly regulate them:

- The “collision of duties” (*Pflichtenkollision*) is a defence *sui generis* (infra III.2.2(3.2.2)), defined as a conflict between two or more grounds of obligation of an equal nature that cannot be cumulatively fulfilled.^23^ Because a decision of the Imperial Court of Justice (*Reichsgericht*) in 1890,^24^ it has been recognized under customary law.^25^ It is regularly applied by the courts; however, it remains highly controversial if the institute excludes the *Tatbestandsmäßigkeit*^26^ or it is used in defence as a justification or excuse.
- Furthermore, the existence of a supra-legal excusing necessity (*übergesetzlicher entschuldigender Notstand*) is discussed in Germany (infra II.2.3.b(2.2.3.b)). It is meant to cover cases beyond the remit of § 34 StGB, because such interests cannot be quantified (such as human lives); nor are they within the remit of § 35 StGB, because the perpetrator is acting in favour of persons other than relatives and close friends.^27^

### Switchman Case (Weichenstellerfall) and the Trolley Problem

#### Outline of the Problem

Dilemma situations were often the subject of theoretical thought experiments. At the beginning of the 20th century, road and rail traffic often played a role in this. As early as 1915, *Kohler* presented the case of an “automobile driver” whose car could no longer be brought to a standstill over a short distance. He could only steer to the right or left, but there were people in front of him, who could not step aside.^28^ Very similar are the German switchman case (*Weichenstellerfall*) by *Hans Welzel* (1951)^29^ and the Trolley problem described by the British philosopher *Philippa Foot* (1967) and further developed by the recently deceased *Judith Thomson* (1985). In *Welzel’s* textbook example,^30^ a freight train approaches a fully occupied standing passenger train owing to a positioning error. A switchman recognizes the danger and diverts the freight train to a siding, so that it races into a group of track workers, all of whom are killed. In the Anglo-Saxon variant, an out-of-control streetcar threatens to roll over five people. Again, they can only be rescued by pulling a lever, so that the trolley will switch to a different set of tracks (however, there is one person on the side-track)^31^ or by pushing an uninvolved “fat man” off a bridge (his massive body would bring the streetcar to a stop).^32^ We see that thought experiments do not necessarily have to represent reality, but rather the structure of a problem.

#### No Justification Under German Law

According to German criminal law, the switchman acts illegally when he diverts the approaching train to another track, so that only a few track workers are killed instead of the many passengers.^33^ Also illegal is the killing of a single person (including *Thomson*’s fat man) to save several other people. Furthermore, in terms of quality, no distinction may be made: the “withered old man” must not be sacrificed at the expense of the powerful young man or the mentally ill person in favour of a Nobel Prize winner,^34^ because each life represents an absolute maximum value.

For example, after the terrorist attacks of September 11, 2001 on the World Trade Center in New York, the question was discussed whether the shooting down of a fully loaded aircraft hijacked by terrorists and intended to be used as an instrument of attack (against a high-rise building or a nuclear power plant) is legally permitted or conversely would lead to severe criminal consequences for the fighter pilot and his superiors, in view of the numerous innocent passengers involved. A German public-law authorization, which would have allowed the German air force to shoot down the plane, was declared void by the German Federal Constitutional Court (*Bundesverfassungsgericht*),^35^ because it was incompatible with the guarantee of human dignity (Article 1, paragraph 1 of the Grundgesetz – GG, i.e., Basic Law) and the right to life (Article 2, paragraph 2, sentence 1 of the GG) guaranteed by the German constitution. Of course, the court clarified that this only applied to people on board who were not involved in the kidnapping, not to the terrorists. Most criminal scholars regard this judgment as decisive for the interpretation of § 34 StGB,^36^ even though the constitutional court expressly emphasized that it did not have to resolve how a shooting that occurred regardless of this finding was to be assessed under the applicable criminal law.^37^ Since then, the discussion has arisen repeatedly in literature and the media.^38^

In US criminal law, where the choice of evils defence is based more on rational expediency, weighing lives against each other does not appear to be excluded from the outset^39^ – in contrast to the *common law* tradition.^40^ The same could possibly apply to the criminal law in countries such as Japan, South Korea, and Taiwan, even if these three jurisdictions have traditionally strong connections to German legal thought. If the conflicting interests are equal, at least the statutory law gives neither a positive nor a negative verdict of illegality. Furthermore, the necessity regulations in Article 37 of the Japanese Criminal Code (緊急避難), Article 22 of the Korean Criminal Code (긴급피난), and Article 24 of the Taiwanese Criminal Code (緊急避難), only provide for the exclusion of criminal liability as a legal consequence. Nevertheless, it is controversial in these countries (according to the German tripartite analysis of criminal law) whether the provisions contain a justification or excuse, or both.^41^

According to the German understanding, the switchman would only act in the sense of a justifying state of necessity (§ 34 StGB) if he perceives an overriding interest; e.g., if he sets the switch in such a way that the train strikes a herd of sheep.^42^

#### Possible Excuses Under German Law

a) The actor can only achieve impunity if he wants to save himself (as with the shipwrecked people) or a close relative (for example, on the train). According to the German understanding, such conflict situations can only be taken into account at the level of culpability: namely, through the necessity defence according to § 35 StGB. Under the terms of the provision, an individual could take a life to save himself or close others from death or serious bodily injury. The guilt is reduced on the one hand because of the special psychic predicament and on the other hand because of a certain reduction of injustice compared with a normal case of homicide (at least one human life is saved).

b) The excusing supra-legal necessity defence (*übergesetzlicher entschuldigender Notstand*) is based on similar considerations: According to the major academic opinion,^43^ it is applied in constellations where the perpetrator (as in the case of the switchman) sacrifices a few people for the benefit of many; the latter need not necessarily be relatives of the perpetrator (as in § 35 StGB). Such an excuse has been previously discussed by the courts in connection with various trials concerning the Nazi euthanasia crimes.^44^ The main focus has been on the accusation of aiding and abetting the murder of mentally ill people in the years 1940–1941 in six killing centers of the German Reich that were specially equipped with gas chambers. Hospitals, nursing homes, and sanatoria were required to report on their patients. In their defence, physicians in psychiatric hospitals who cooperated and were later indicted claimed that by extraditing a few patients, they had prevented something worse (namely, the killing of all inmates of the institution). However, in many cases the alleged dilemmas turned out to be refutable lies.^45^ Insofar as an actual conflict for the physicians could not be ruled out, the German higher courts occasionally reached a “personal reason for exclusion from punishment” decision in the immediate post-war period (1945–1949).^46^ However, the Federal Supreme Court (BGH) made no such decisions after 1949.^47^

Therefore, the preconditions for a supra-legal necessity defence are largely unclear. The shooting down of a passenger aircraft used as an instrument of attack (in a “9/11-scenario”, supra II.2.2(2.2.2)) is a possible future precedent that has not been excluded by the Federal Constitutional Court (*Bundesverfassungsgericht*).^48^

#### Interim Conclusion

It makes a difference whether the perpetrator acted in accordance with the law or merely had been discharged for lack of personal culpability. Despite the exclusion of culpability (supra II.2.3(2.2.3)), the potentially injured are not subjected to an unreasonable duty to acquiesce in either case constellation (their right of self-defence is not lost),^49^ which would be the case if the perpetrator was justified. Three arguments seem to be decisive for the solution of *Welzel*’s switchman case and *Foot*’s Trolley problem according to German criminal law:

*Argument 1:* Every human life has the same rank and eludes any quantification. No one can be expected to sacrifice himself in favour of one or more others.

*Argument 2:* Action is more significant than mere omission. The active killing of a person weighs so heavily that even the rescue of a large number of people could never compensate for this. Prohibitions have to be respected over commands.

*Argument 3:* Secured legal positions (standing on a side-track without danger) enjoy a higher level of protection than mere expectances (the chance to be rescued by changing the switch).

This is almost logically compelling. Otherwise (to take an example from US literature), in extreme cases one could force every healthy person to sacrifice himself to save the lives of at least five seriously ill patients with his transplantable organs (heart, lungs, two kidneys, and one liver).^50^ Such a cruel duty of solidarity^51^ cannot exist and is unlikely to be advocated even by the greatest utilitarian. In Germany, it would be incompatible with the guarantee of human dignity of Art. 1 I Grundgesetz (“human dignity shall be inviolable”, supra II.2.2(2.2.2)). The violation of such elementary fundamental rights cannot be justified by advantages for third parties.

## Transferability of These Principles to the Triage?

### Outline of the Problem

It is now questionable how the aforementioned principles can be applied to the problem of triage in the context of COVID-19. In addition to the numerous statements from criminal law scholars,^52^ the German Ethics Council^53^ and national medical societies^54^ have also issued recommendations. According to these recommendations, the decision as to who should be given priority treatment should be made according to the “two-man rule,” with the participation of two doctors experienced in intensive care medicine.^55^ In the prioritization process, not the age of the patient (Italy and Switzerland are more outspoken),^56^ but the chances of success of an intensive care therapy should play a role; these are considered poor if no improvement or stabilization is to be expected or survival would be tied to permanent confinement in an intensive care unit.^57^

However, the problem of course remains primarily a legal one.^58^ Recommendations of the Ethics Council and professional associations must be measured against the applicable law: In December 2021 the German Constitutional Court decided, that it would be against Art. 3 (3) 2 Grundgesetz (Basic Law) disadvantaging patients based on disability.^59^ In November 2022 the German legislator made changes in the Infektionsschutzgesetz (Protection against Infection Act)^60^. According to the new § 5c Infektionsschutzgesetz an allocation decision may be made on the basis of the current and short-term survival probability of the patients concerned, but not on the degree of infirmity, age, ethnic origin, religion or belief, gender or sexual orientation. However, age, frailty due to age or disability are overwhelmingly considered to be specific risk factors for a negative short-term prognosis of patients in intensive care. So it is questionable, how physicians should deal with these guidelines.^61^ Furthermore, the new regulations only apply in the context of pandemics, but not in the case of natural disasters, war or terrorist attacks.

However, also substantial criminal offences have to be considered: a physician who refuses to provide medical treatment or who abruptly ends intensive therapy to treat another patient may be punished for intentional homicide (§ 212 StGB) or causing bodily harm (§ 223 StGB) by omission (§ 13 StGB) if the patient dies as a result, suffers unnecessary pain, or suffers damage to health. While in the case of the switchman (supra II.2.1(2.2.1)), duties to act and duties to omit collide and the perpetrator is reproached for his actions; in this context, it is primarily a matter of colliding duties to act. However, an accusation of punishable inaction can only be made against the physician if he has the opportunity to provide the treatment. We can distinguish between two different cases:

*Case 1:* Two ventilation places are available in a hospital. Five patients arrive at the same time, including a 20-year-old vaccine refuser who further ignored all travel warnings, a vaccinated 35-year-old family man with two small children, a 55-year-old doctor who became infected by a new dangerous variant while treating COVID-19 patients, and two elderly people. All five need ventilators. The short-term survival probability of the patients cannot be accurately answered.

*Case 2:* All ventilation places are occupied by other patients, including an 83-year-old man with significant pre-existing conditions. Now a 35-year-old father of two small children who also needs to be ventilated urgently is admitted to the clinic. His chances of survival would be much better.

### Ex ante Competition (Case 1)

#### Possible Grounds for Exclusion, Justification, and Excuse

In *case 1*, five equivalent duties to act collide, of which the physician can fulfil only two. First, *Argument 1* (supra II.2.4(2.2.4)) that every human life has the same rank also applies here without compromise. In the case of conflicting duties, § 34 StGB (justifying necessity) does not apply, because the life of one patient does not outweigh that of the other (supra II.2.2(2.2.2)), nor does § 35 StGB (excusable necessity) apply unless the responsible physician acts in favour of a relative (supra II.2.3.a (2.2.3.a)). However, the nonstatutory institute of the collision of duties (*Pflichtenkollision*, supra II.1.2.a (2.1.2.a)) must be considered if one assumes that the respective need for treatment is equally urgent^62^ and all patients want to be ventilated by machines. Especially with older patients this is not a matter of course, because ventilation via a plastic tube inserted into the trachea can be quite painful.

#### Legal Classification of the Conflict of Duties as a Defence Sui Generis

According to the main academic opinion, the collision of duties (*Pflichtenkollision*) in German criminal law is not a mere excuse.^63^ This is absolutely convincing. After all, impunity is not based on the perpetrator’s lack of insight or control. A mere excuse would also have the consequence that the patients or their relatives would have to be granted the right of self-defence or emergency aid (they could force the doctor to come to the aid of their relatives first).^64^ Hospital violence is not a completely unrealistic scenario^65^ and would ultimately block any rescue.^66^

The major opinion^67^ in Germany assumes that the collision of duties is a justification. In my opinion, the nonstatutory *Pflichtenkollision* should even exclude the objective side of the offense.^68^ Because it is not the principle of predominant interest that is applied here, which is actually typical for justifications, but the principle already known in Roman law, that the impossible cannot be demanded by the legal system – *impossibilium nulla est obligatio*.^69^ For a lawyer from a common law jurisdiction traditionally used to rather equally ranked defences, this discussion may be weird. Real differences in criminal liability – to be honest – do not result from the one or the other concept (at least at first glance). In each case, it is sufficient that the person responsible fulfils part of the conflicting obligations to be found not guilty. However, by placing the conflict within the objective elements of the offense, it is made clear that the death of three patients cannot be attributed to the doctor as “his work” – the patients died of a fatal illness rather than medical negligence or even malice.^70^ Furthermore, the rescue of two patients with the sacrifice of the other three is not connected to a positive legal judgment, which seems also unreasonable. Nature acts in a morally neutral manner.

#### Applicability of the Collision of Duties in Case 1 (“Ex Ante Triage”)

In *case 1,* the physician can only provide necessary treatment to two of five patients. Then it must be up to him – regardless of the aforementioned dispute – to decide which commandment he wants to fulfil.^71^ The law does not offer any clear positive selection criteria for this decision.^72^

The physician may decide on the basis of prospects for short-term clinical success (supra III.1(3.1)). However, if this question cannot be accurately answered (what is quite probable), he maybe uses other criteria, such as individual responsibility, (long-term) life expectancy,^73^ marital status, and the social value of the profession.^74^ Thus, he could put the 20-year-old vaccine refuser and returning traveller (despite his youth) in second place owing to his irresponsible behaviour,^75^ even though it is debatable whether the denial of basic health and life chances is really appropriate in these cases.^76^ He could give preferential treatment to the family man (because of his parental responsibilities) or to the doctor (on the principle of “save the rescuers”),^77^ because it is expected that after his recovery (and immunization) he will be able to participate particularly effectively in the fight against the pandemic. He could decide by lot also.^78^

As long as the criminal law cannot clearly determine for which patient the guarantor is to decide (the new § 5c Infektionsschutzgesetz does not either)^79^, subsequent disapproval of the behaviour is not possible.^80^ The recommendations of the professional associations, which pursue the ethically convincing goal of saving as many patients as possible with the available resources, have no legal force.^81^ A deviation cannot have consequences in terms of criminal law (but may do under labour law).^82^ Also the new § 5c Infektionsschutzgesetz is not meant to change the criminal assessment, at least according to the reasoning in the draft bill.^83^ In my opinion the uncountable value of every human life (supra II.2.2 (2.2.2) and II.2.4 (2.2.4)) ensures the impunity of the physician even if he saved the life of a patient who had somewhat lower chances of survival than another patient who does not receive treatment.^84^

### Ex post Competition (Case 2)

*Case 2* is even more difficult to assess. The recommendations of the professional associations probably assume that an ongoing treatment that is still indicated could be terminated, because the ventilation place is needed for a patient with a greater chance of survival. In any case, all patients requiring intensive care should be considered equal when prioritizing.^85^ According to the guidelines of the Swiss Academy of Medical Sciences, the continuation of treatment should also be checked every 48 hours on the basis of a list of clinical criteria.^86^ According to British recommendations, health professionals may be obliged to withdraw treatment from some patients to enable treatment of other patients with a higher chance of survival. They argue that there is no ethically significant difference between decisions to withhold life-sustaining treatment or to withdraw it, other clinically relevant factors being equal, although health professionals may find decisions to withdraw treatment more challenging.^87^ The recommendations of the German Ethics Council are more reserved in this respect. The termination of a life-sustaining clinical procedure may be illegal, even if it follows ethical and transparent criteria.^88^

#### Possible Reasons for Exclusion and Justification

It is questionable whether it is legal to disconnect the 83-year-old patient who is still ventilator-dependent from the device to treat subsequently the 35-year-old father of a family with two small children, because the latter has better chances of survival owing to his age and loses more years of life if he is not ventilated. The German draft for a new § 5c Infektionsschutzgesetz (Protection against Infection Act) states, that already allocated intensive care treatment capacities should be excluded from the allocation decision.^89^ If one regards the separation as a positive act, then one will have to realize, analogous to *Welzel*’s switchman case (supra II.2.1(2.2.1)), that the active killing of a human being (§ 212 StGB) for the purpose of saving the life of another can never be justified by § 34 StGB.^90^ Of course, the collision of duties also must be considered (supra III.2.2(3.2.2)). However, according to *Argument 2* (supra II.2.4(2.2.4)), duties to provide assistance are generally regarded as less important than the prohibitions of causing injury. The fact that the separation of the patient from the ventilator is associated with the use of energy could be an argument against its application.

#### Applicability of the Collision of Duties in Case 2 (“Ex Post Triage”)

However, it would also be conceivable that the doctor's behaviour can be reinterpreted as an omission, as the focus of the legal reproach may not be switching off the device, but failure to continue treatment of the 83-year-old patient.^91^ In neutralizing *Argument 2* (supra II.2.4(2.2.4)) by means of such a dogmatic “trick”, it does not seem impossible from the outset to achieve impunity through the institute of collision of duties (supra III.2.2(3.2.2) and III.2.3(3.2.3)). In fact, one could argue that there is no real qualitative difference from mouth-to-mouth resuscitation in the context of immediate life-saving measures (the termination would undoubtedly have to be considered an omission). In addition, a ventilator could be technically designed in such a way that it would need a positive impulse every hour to continue the ventilation activity.^92^ On the other hand, the act of withdrawing a ventilator may be connected with further actions (e.g., waking up the patient so that his respiratory reflexes can kick in). Nevertheless, the punishability of the physician cannot really depend on the rather arbitrary and situation-dependent evaluation of his behaviour as acting or omitting to act.^93^

Decisive for the solution of *Welzel’s* case was probably less the distinction between doing and not doing as such, but rather *Argument 3* (supra II.2.4(2.2.4)), that secured legal positions (to stand on a siding without danger) as the given status quo enjoy greater protection than mere expectances (the chance of the train passengers being rescued by changing the switch).

Here, the 83-year-old, who is already being ventilated, has such a secured legal position. With the connection to the ventilator, he has left the group of those competing for a place on the ventilator, and he or his relatives will have ceased looking for an alternative hospital, trusting in the position they have attained (comparable to a passenger who has found a place in a lifeboat after a shipwreck).^94^ An intervention in this secured legal position is only permitted for the purpose of safeguarding much higher-value interests, which is not the case if the maximum value of life is affected on both sides (cf. *Argument 1,* supra II.2.4(2.2.4)). This thought also prevails if one assumes that the doctor will not continue the treatment in this case.^95^ There is no equivalence of conflicting duties. A different decision can only be made if the condition of the 83-year-old patient receiving initial treatment has deteriorated to such an extent that further treatment appears hopeless (in which case further treatment is no longer indicated), or conversely the risk of death has been reduced to the level of a mere abstract danger to life.^96^

### Interim Conclusion

As a result, in *case 1,* the physician may decide which patients will receive treatment, if one assumes that the respective need for treatment is equally urgent. According to the criminal law, he can, but does not have to, follow the recommendations of the medical societies. It is an unsolved question, if the criteria of § 5c Infektionsschutzgesetz are binding in a criminal law sense. In my opinion, even in the case of (illegal) discriminatory or corrupt allocation decisions, offences against the person (like homicide) seem to be unsuitable for punishing such violations of the law.^97^ Also in this regard the uncountable value of every human being has to be taken into consideration.

In *case 2,* the physician should not terminate the treatment of the 83-year-old patient (against the recommendations of the professional associations). A defence can only be considered in the case of § 35 StGB, if the physician acts in favour of a relative (supra II.2.3.a(2.2.3.a)). No excusing supra-statutory necessity (supra II.2.3.b(2.2.3.b)) has yet been explicitly recognized by the courts, and even the criteria established by the literature are not met, because it is not a matter of sacrificing a few in favour of saving many. Even an unavoidable error juris according to § 17 sentence 1 StGB (a defence in Germany)^98^ will hardly be accepted,^99^ because the medical societies themselves admit that the termination of intensive care measures in the context of prioritization in Germany may “come up against legal limits”.^100^

## Transferability of These Principles to Emergency Algorithms for Autonomous Vehicles

### Outline of the Problem

1. The aforementioned principles can also be applied to conventional road traffic. For example, a human driver who deliberately steers his vehicle into a crowd of people instead of against a deadly obstacle that surprises him is acting illegally (in the sense of the tripartite analysis of criminal law in II.2.1, above). However, he will not be punished for manslaughter. The conflict situation of the individual to have to sacrifice himself must be taken into account (as with a shipwrecked person) on the level of culpability, namely by § 35 StGB (supra II.2.3.a(2.2.3.a)). Even if many lives could be saved, the danger to certain road users must not be averted in an inadmissible way at the expense of another road user^101^ – analogous to *Welzel*’s switchman case (supra II.2.1(2.2.1)).

Therefore, the driver of a fully occupied school bus is not justified in (but may be excused for) preventing a collision with a surprising obstacle that would be fatal for all passengers by intentionally (but regretfully) running over a pedestrian on the sidewalk.^102^ However, in most real-life dangerous situations, the human driver has hardly any time for lengthy deliberations; he will often make some decision “reflexively” – with no chance to weigh up the pros and cons. Thus, prosecution is unlikely to occur, unless the driver’s negligence caused the dilemmatic situation.

2. Many German authors^103^ want to transfer the aforementioned principles (unchanged) to autonomous vehicles. Of course, an autonomous vehicle must always favour material damage over personal injury; this is not controversial. However, according to these authors, it would be illegal to program a “utilitarian” steering impulse that saves many people at the expense of an innocent pedestrian on the sidewalk. The reasoning is based on the above principle that a danger to certain road users resulting from fateful circumstances should not be averted in an inadmissible way at the expense of another road user (supra IV.1.1.(4.1.1). However, according to this opinion, even programming a “utilitarian” steering impulse would probably be culpable,^104^ as the programmer, developer, management, and other parties are not in an acute mental predicament, when they act. Hence, it does not seem improbable that they could be prosecuted for manslaughter (§ 212 StGB). No owner, manufacturer, or programmer would wish to be exposed to such risks.^105^

Car manufacturers could program the vehicles in such a way that in a dilemma, humans would take over. Of course, this would not be a great improvement; quite the contrary. The time would usually be insufficient for a meaningful decision. The installation of a random number generator has been suggested,^106^ but the programming of a nondeterministic random number generator is not easy. The computer cannot generate real random numbers at all; it always requires the inclusion of external (for example, physical) processes.^107^ Moreover, of course, the decisions are not better.

One could also try to adapt the technology according to the aforementioned academic opinion. However, this will only succeed with great difficulty. How can a machine in a dilemmatic constellation let fate take its course? After all, it was programmed beforehand for every conceivable case. The idea is probably that the autonomous vehicles should brake in an emergency event, but steer straight ahead killing the people in front of them (no matter how many). However, there is no “natural” route that the vehicle would have to take.^108^ On a curve, for example, it is not even clear what “straight ahead” means:^109^ Should the car continue to follow the so-called “vehicle trajectory” or should centrifugal force carry it forward into the “field”?

3. In contrast, the vast majority of the test participants in the *Moral Machine* allow the quantitative weighing of human lives.^110^ The majority also showed a strong tendency to save children rather than older people (although there are cultural differences in this respect) and judged according to whether the persons concerned have followed traffic rules. One could argue that at least US participants tend to orientate themselves more toward their own penal laws (II.2.2(2.2.2)). However, the continental European participants did not achieve a significantly different survey result on this point either. Is there a striking gap between the law and the moral perceptions of citizens?

### Novelty of the Dilemma Problem in Autonomous Driving

The legal situation is not as clear as it seems at first glance; moreover, alternative solutions are increasingly being discussed in Germany.^111^ In my opinion, the dilemma problem in the context of autonomous driving is a new one and can only be compared to a very limited extent with the scenarios discussed so far. The actual decision on how the vehicle should behave is not made at the moment of the accident or immediately before it.^112^ Rather, it is made months or years earlier, in view of a possible (by no means safe) hazardous event with unknown participants.

Of course, one cannot shake the principle behind *Argument 1* (supra II.2.4(2.2.4)) that every human life always has the same value and evades any quantification. Nevertheless, even if human life is always equal, the claim that the killing of the track workers (supra II.2.1(2.2.1)) or the passer-by on the sidewalk (supra IV.1.1 (4.1.1)) is worse than the death of many more passengers on the train or bus also requires justification.

In *Welzel*’s switchman Case, the difference between positive and negative duties played an important role: According to *Argument 2* (supra II.2.4(2.2.4)) the duty to act (to save the lives of the train passengers) is classified as less important than the duty to refrain from killing a track worker, although on both sides the maximum value of life is affected. The human driver in an acute traffic situation may decide only to brake and leave the position of the steering wheel unchanged, letting fate take its course. However, when programming a command (braking yes/no; steering impulse yes/no), we are always dealing with an action, regardless of whether the algorithm lets the car continue to roll “straight ahead” (whatever that means; supra IV.1.2 (4.1.2)) or steers it to the right or left in the case of an impending accident.^113^ Similar to *case 2* of the COVID-19 Triage case (supra III.1 (3.1), III.3.1 and 2 (3.3.1, 3.3.2)), the distinction between doing and not doing as such does not lead to clear results. The option to do nothing, to let fate take its course, is not left to the developer, as long as he continues his work.

In *Welzel*’s switchman case (supra II.2.1(2.2.1)) and *case 2* of the COVID-19 Triage (supra III.3.1 (3.3.1)) problem, there was another important point: According to *Argument 3* (supra II.2.4(2.2.4)), a secured legal position (to stand on a siding without danger or to be ventilated) enjoys greater protection than mere expectances (the chance of the train passengers to be rescued by changing the switch or the chance of the 35-year-old father to receive the ventilator of the 83-year-old patient). An intervention in the secured legal position would only be allowed to safeguard much higher-value interests, which is not the case if the maximum value of life is affected on both sides.

However, in the case of the autonomous vehicle, no one has a secure legal position at the time of programming. It would hardly be possible to foresee the role a certain person may find himself in a potential accident (in the role of the lonely pedestrian who is run over, but perhaps also as part of a group of people who are saved by the pre-programmed steering impulse). As a result, there are no compelling legal reasons for privileging one or the other potential victim at the time of programming (similar to *case 1* of the COVID-19 Triage, supra III.2.3(3.2.3)).

### Conceivable Ethical Objectives

The dilemmatic constellations should ideally be (or be able to be) resolved in such a way that is most acceptable to the population. The results of the *Moral Machine* are an important indicator (supra IV.1.3 (4.1.3)). The German Federal Ministry of Transport and Digital Infrastructure established a commission for the further clarification of “ethical” questions, under the leadership of the former Federal Constitutional Court judge *Udo Di Fabio*. The commission definitely excluded the qualification of people according to personal characteristics (age, sex, physical or mental constitution) in unavoidable accident situations.^114^ A “general programming to reduce the number of personal injuries” could “be acceptable”, whereby (consciously) it remains unclear to which situations it may refer.

In my opinion, regardless of specific participants, it is obvious that the benefits of new technology must be maximized in such a way that the greatest possible number of people have the chance to be spared. Therefore, the degree of imminent danger must also play a role, but not the gender or age of the persons concerned, and certainly not the profession or the social significance. Failure to take self-protection measures (such as the use of a bicycle helmet) could either favour or oppose the person in traffic; the increased risk of injury indicates a greater need for protection, but this might create false incentives to refrain from self-protection measures.^115^ However, in any case, one must assume that the cause of the dangerous situation (a group of drunk people running wantonly across the street) or contributory negligence by the actors must be weighted negatively. It seems unfair to impose the costs of risky misconduct on a (lonely) uninvolved person who has done nothing wrong himself.^116^ Only with respect to children – particularly endangered in road traffic (and until the 10th year of life not responsible under German civil law, § 828 II 1 BGB) – should this point not apply.

Car manufacturers will also ask themselves whether they are allowed to give vehicle occupants a preferential position.^117^ One argument for this could be that human drivers generally do not behave differently; i.e., they instinctively protect themselves and their passengers at the expense of others (supra IV.1.1 (4.1.1)). Therefore, the society does not lose anything. If purely utilitarian programmed vehicles were to compete with those that guarantee increased self-protection (even at the expense of others), the former would have little chance of selling on the free market.^118^ However, the argument against permissibility is that it is specifically the vehicle’s occupants who will benefit most from the new technology. It would appear unfair for them to pass the residual risks to outsiders or even optimize them. Of course, passive systems that mitigate the consequences of an accident for the occupants are unproblematic.

### Applicability of the Collision of Duties

These aforementioned ideas (somewhere between *Kant* and *Bentham*)^119^ must of course be measured against the applicable criminal and constitutional law. In my view, the programmer of an emergency algorithm sees himself confronted by a collision of duties to omit (thus conflicting prohibitions) rather than by a duty to omit and a duty to act (from which the opposite view proceeds, supra IV.1.2 (4.1.2)). A collision of duties to omit has hardly been discussed in jurisprudence so far. This is no coincidence. Real collisions of equivalent prohibitions seldom occur in real life, because prohibitions normally do not completely exhaust the sphere of action. Some academics^120^ even claim that they do not exist from a logical point of view, quoting the jurist, mathematician, and philosopher *Christian Wolff* (1679–1754):

“*Leges prohibitivae nunquam inter se colliduntur*”—prohibition laws never collide with each other.^121^

I would not follow that argument. In most cases, of course, we face avoidable state failure. Imagine, after a change in traffic routing, four one-way streets suddenly meet. There must be some way out for the driver, who is virtually “trapped” at the crossing. In my opinion, it is not illegal if he leaves the “trap” against the direction of travel via one of the one-way streets. Why should the perpetrator not be granted impunity and freedom of choice (supra III.2.3 (3.2.3)) when he is confronted with equal prohibitions?^122^ It is generally accepted that a legal system must not demand the impossible from anyone (supra III.2.2(3.2.2)).

Two other examples may be more appropriate in this context. In a mass panic (such as during religious pilgrimages or large entertainment events or concerts) a person who is pushed forward by a crowd of people may find himself in the situation of having to step either on A or on B, who have fallen in front of him.^123^ It would be wrong to regard his behaviour as a deliberate, illegal attack. No matter what the offender decides, the injury to A or B remains an accident. The same must apply to an airplane crash where the pilot has only the choice to steer the plane into one (frequented) or another (less frequented) building. In a situation in which the perpetrator *must* actually do something*,* the law must not deny him every opportunity to act (whereby in my opinion the objective side of the offense should be excluded, supra III.2.2(3.2.2)); everything else would amount to an invalid “norm trap”.^124^

Nevertheless, the constellation when programming emergency algorithms deviates on one point from the abovementioned cases: While the perpetrator in the crowd and the pilot must act in some way, the programmer could completely decline to cooperate in the development of vehicle algorithms. Of course, nothing would be gained by this. In 2021, there were 2.562 traffic-related deaths in Germany and 42,915 in the US; misbehaviour by human beings is considered the cause in 88.0% of the accidents.^125^ The technology of autonomous cars has the potential to save a considerable number of lives.

### Social Adequacy and Risk Minimization

As we have already seen, the programming of algorithms is not performed for people in actual emergency situations but with a view to a possible dangerous event with unknown participants (supra IV.2 (4.2)). Here, no one presumes to play “destiny” (or even God) for certain people, in stark contrast to *Welzel*’s switchman case (supra II.2.1(2.2.1)) or to some physicians in the Nazi era who allegedly wanted to prevent worse things by “sacrificing” a few patients (supra II.2.3.b (2.2.3.b)).

Loss of life on the road has always been accepted; this is not an innovation of autonomous driving. According to the doctrine of social adequacy, actions that appear to fulfil all elements of a crime but are entirely within the normal social order do not constitute injustice. The taking of risks is allowed if every average citizen agrees to the extension of freedoms in the hope of being spared from the risks.^126^ In the case of negligence, the permitted risk is the flipside of a breach of due diligence. Under road traffic law, once a vehicle has been registered its distribution and operation are within this framework, even though the manufacturer and the owner can of course never rule out the possibility of an accident involving personal injury. Danger or harm resulting from these risks is not imputable to the actor despite the existing causal link between his conduct and the damage or injury.

Another question is whether this can be transferred specifically to the programming of emergency algorithms. Here we have to think in the categories of an intentional offense. In German criminal law, intention is the label used not only for cases of direct intention (*dolus directus I*) and almost certain knowledge (*dolus directus II*), but also includes cases of “conditional” intention (*dolus eventualis*). German law has no category of *mens rea* that fully equals the concept of recklessness as understood in common law.^127^*Dolus eventualis* means that the actor considers the realization of the injury as possible and accepts it if it should occur. If the offender hopes it will not materialize, he only acts with conscious negligence (*luxuria*). However, even if the probability of an accident is very low, the emergency algorithms are programmed for this very case. It will be hard to deny that the developer is willing to accept that a causal chain is set in motion and causes the death of one or more people. Can one still speak of a permitted risk here?

I think it is possible, as his programming is a step to minimize the abstract danger of the car for everyone, which seems to me to be the crucial point. For the track workers (supra II.2.1(2.2.1)), the decision of the switchman at the time of his act was undoubtedly disadvantageous. On the other hand, from an *ex ante* point of view*,* in automated road traffic every single member of society benefits from more people receiving a certain amount of preferential treatment, and behaviour that conforms to the rules is rewarded (supra IV.3 (4.3)). The general reduction in the number of accidents and victims ultimately lowers the probability of becoming a victim.^128^

A programmer who reduces the hitherto accepted risk of an individual becoming the victim of a traffic accident, while at the same time having no better choice as the result of exposure to conflicting duties (supra IV.4 (4.4)), does not create a legally disapproved danger. If the assessment turns out to be wrong to certain persons in retrospect, it does not matter. Wearing a seat belt can also be disadvantageous in about 1% of accidents (for example, in the event of a fire or a fall into water),^129^ but in many more cases, the obligation to wear a seat belt saves lives.^130^ This approach can also be justified from the perspective of constitutional law and deontological moral philosophy. The individual is not only an “object” or “tool,” as *Hevelke* and *Nida-Rümelin* correctly describe it, but also the “purpose” of such programming.^131^ This is the difference between the accident victim, who (unfortunately) has become the target of the algorithm, and the track workers or aircraft occupants (supra II.2.2(2.2.2)), who are purposefully sacrificed for others, as well as the healthy person asked to give up his life to save five seriously ill people (supra II.2.4(2.2.4)). Thus, the guarantee of human dignity (article 1 paragraph 1 GG) remains untouched (supra II.2.2(2.2.2)). The privileging of specific groups (old or young people, men or women), which would go hand in hand with a general increase in the risk to others, would certainly not be compatible with this approach, but it would also be undesirable from a legal policy standpoint.

### Interim Conclusion

An algorithm that reduces the *ex ante* risk of each individual becoming the victim of an accident without creating false incentives does not impose unreasonable solidarity obligations on anyone, even if it proves to be disadvantageous for individuals in retrospect. However, the issue remains highly controversial in Germany at present. The solution proposed by opponents (supra IV.1.2 (4.1.2)), which is closely based on the classic textbook scenarios with human decision-makers, leads to results that are unsatisfactory and difficult to implement in practice. The principle that duties to help are less important than prohibitions of infringement is correct; however, it is not applicable in this context. The analogy to *Welzel*’s switchman case fails to recognize that at the time of the accident, the developer has no control options whatsoever, but is active in programming, regardless of whether the preliminary decision he makes leads to a new steering impulse or to remaining on the previous “vehicle trajectory”.

## Conclusion

The question of how to deal with dilemma scenarios was discussed in antiquity; it fills libraries. It has also been the subject of the largest cross-cultural study of moral preferences ever since. In most of the debated cases, the death of one or more people is absolutely unavoidable. The protagonists have regularly not caused the situation themselves, but fatefully came into the conflict. Even a person who did everything right in the end will have to struggle with regrets and feelings of guilt (“moral residue”). Some researchers of course, have criticized the use of stylized dilemma scenarios, like *Welzel*’s switchman case or the Trolley problem, arguing that the scenarios are too extreme and unconnected to real-life moral situations.^132^

However, the legal problem of prioritizing medical aid, which had previously also been rejected as theoretical, suddenly became real in 2020 in connection with the COVID-19 pandemic (even in the Western world). The *ad hoc* recommendations of the European professional associations expose doctors to unnecessary criminal liability risks, as withdrawing life support from one patient to provide it to another could be assessed as a homicide. Therefore, a clear warning from the legal professionals was urgently needed. Ethical conflicts also arise in engineering; the solution to the dilemma of who must die in an unavoidable accident seems to be one of the last unresolved legal problems of autonomous vehicles. Even though the automotive industry promises that such situations will hardly ever occur, it could prove to be a tangible obstacle to acceptance and innovation. Nevertheless, the cultural differences in judging such scenarios are not as high as they appeared at first glance. The programming of an algorithm with regard to a possible accident at some point in the future, with participants still unknown, differs significantly from *Welzel*’s switchman case and *Foot*’s trolley problem. The goal of saving as many lives as possible to minimize the chances of anyone involved in a grave accident seems to be widely accepted. In Germany, the dilemma problem is also legally solvable in a satisfactory way (between utilitarian and deontological moral principles), although emergency algorithms may lead to a certain shift in the previously accepted risk.

In the end, we may ask ourselves why philosophy professors and law teachers are so “fascinated” by such dilemmas. To be honest, it is not primarily to provide practical legal guidelines for doctors or the automotive industry, who must not be left alone with their “worries and needs.” Of course, the law-abiding citizens want and have the right to know right from wrong. However, the discussions also have an end in itself. Moral and criminal law systems must be free of contradictions. There is no legal or moral question that can remain unanswered. When weighing up life against life, our systems are put to a final test. The debated problems are understood (and can arise equally) in all countries around the world. They prompt a strong plea for a more global criminal law theory; however, this should not be limited to solving distinct stylized cases but should take into account the underlying criminal law concepts.^133^ For example, the tripartite analysis of German criminal law (the distinction between the statutory elements of the offense, the wrongfulness and the culpability) – its strengths and weaknesses – could perfectly be exemplified by the dilemma scenarios discussed here.

## Footnotes

1 Cf. Markus D. Dubber, *Theories of Crime and Punishment in German Criminal Law*, 53 The American Journal of Comp. Law 679 (Summer 2005); Hans-Heinrich Jescheck, *The Significance of Comparative Law for Criminal Law Reform*, 5 Hastings Int'l & Comp. L. Rev. 1, 17 et seq. (1981). In German criminal procedure law, in contrast, Anglo-American models were repeatedly adopted (e.g., the idea of a “fair trial” and the practice of plea bargaining).

2 The Hungarian–British psychoanalyst Michael Bálint describes the phenomenon as a “thrill” (English) or “Angstlust” (German).

3 The story is attributed to the Greek philosopher Carneades of Cyrene (214–129 BC) and may be found for example in Cicero’s *De Officiis* 3.89. The example was taken up by the German philosophers, e.g., Samuel v. Pufendorf, *De Iure Naturae Et Gentium Libri Octo*, Lib. II, Cap III, § 11 (1754); Immanuel Kant, *Die Metaphysik der Sitten, Erster Theil, Metaphysische Anfangsgründe der Rechtslehre,* Anhang II zur Einleitung in die Rechtslehre (Das Nothrecht), p. XLI (1798).

4 For example, in Claus Roxin & Luís Greco*, Strafrecht Allgemeiner Teil I*, § 22 no. 16 (5th ed. 2020).

5 United States v. Holmes, 26 F. Cas. 360 (1842).

6 R. v. Dudley and Stephens, 14 QBD 273 DC (1884).

7 Cf. Gustav Radbruch, *Der Geist des englischen Rechts*, pp. 87 et seq. (1946); Hans-Heinrich Jescheck & Thomas Weigend*, Lehrbuch des Strafrechts: Allgemeiner Teil*, § 21 I (5th ed. 1996); Roxin & Greco, supra note 4, § 16 no. 35.

8 The decision is still considered authoritative in England and Wales. However, see also Re A (conjoined twins) 2 WLR 480 (2001) on the separation of Siamese twins, which was considered acceptable.

9 For example, Hauke Brettel & Jan Schuhr, *Triage und Priorisierung*, Medizinrecht (MedR) 40, 801 (2022); Armin Engländer & Till Zimmermann, “*Rettungstötungen*” *in der Corona-Krise?,* 2020 Neue Juristische Wochenschrift (NJW) 1398; Karsten Gaede & Michael Kubiciel & Frank Saliger & Michael Tsambikakis, *Rechtmäßiges Handeln in der dilemmatischen Triage-Entscheidungssituation*, 2020 Medizinstrafrecht (medstra) 129; Thomas Gutmann & Bijan Fateh-Moghadam, *Geplante Regelung der Triage – Grundrechtsschutz als Farce*, 2022 ZRP 130; Tatjana Hörnle, *Dilemmata bei der Zuteilung von Beatmungsgeräten*, Verfassungsblog Apr. 4, 2020 (on the Internet at https://verfassungsblog.de/dilemmata-bei-der-zuteilung-von-beatmungsgeraeten/); Elisa Hoven, *Die* “*Triage*”*-Situation als Herausforderung für die Strafrechtswissenschaft,* 2020 JuristenZeitung (JZ) 449; Christian Jäger & Johannes Gründel, *Zur Notwendigkeit einer Neuorientierung bei der Beurteilung der rechtfertigenden Pflichtenkollision im Angesicht der Corona-Triage*, 2020 Zeitschrift für Internationale Strafrechtsdogmatik (ZIS) 151; Merkel & Augsberg, *Die Tragik der Triage – straf- und verfassungsrechtliche Grundlagen und Grenzen,* 2020 JZ 704, 708; Wolfgang Mitsch, *Bemerkungen zur „präventiven“ Triage und zur „ex-post“-Triage*, 2022 Zeitschrift für Internationale Strafrechtswissenschaft (ZfIStw) 323; Thomas Rönnau & Kilian Wegner, *Grundwissen – Strafrecht: Triage*, 2020 Juristische Schulung (JuS) 403; Christoph Sowada, *Strafrechtliche Probleme der Triage in der Corona-Krise*, 2020 Neue Zeitschrift für Strafrecht (NStZ) 452; Detlev Sternberg-Lieben, *Corona-Pandemie, Triage und Grenzen rechtfertigender Pflichtenkollision*, 2020 Medizinrecht (MedR) 627; Jochen Taupitz, *Verteilung medizinischer Ressourcen in der Corona-Krise: Wer darf überleben?*, 2020 Medizinrecht (MedR) 440; see also the two anthologies of Eric Hilgendorf, Elisa Hoven & Frauke Rostalski (eds.), Triage in der (Strafrechts-)Wissenschaft (2021) and Tatjana Hörnle & Stefan Huster & Ralf Poscher (eds.), Triage in der Pandemie (2021).

10 For example, Armin Engländer, *Das selbstfahrende Kraftfahrzeug und die Bewältigung dilemmatischer Situationen*, 2016 ZIS 608; Sabine Gless & Ruth Janal, *Hochautomatisiertes und autonomes Autofahren,* 2016 Juristische Rundschau (JR) 561; Tatjana Hörnle & Wolfgang Wohlers, *The Trolley Problem Reloaded*, 2018 Goltdammer’s Archiv für Strafrecht (GA) 12; Jan C. Joerden, *Zum Einsatz von Algorithmen in Notstandslagen* in *Autonome Systeme und neue Mobilität*, pp. 73 et seq. (Hilgendorf Ed., 2017); Frank Peter Schuster, *Das Dilemma-Problem aus Sicht der Automobilhersteller – eine Entgegnung auf Jan Joerden*, *ibid*., pp. 99 et seq.; Frank Peter Schuster*, Notstandsalgorithmen beim autonomen Fahrzeug,* 2017 Recht Automobil Wirtschaft (RAW) 13; Frank Peter Schuster, *Strafrechtliche Verantwortlichkeit der Hersteller beim automatisierten Fahren*, 2019 Deutsches Autorecht (DAR) 6, 9 et seq.; Thomas Weigend, *Notstandsrecht für selbstfahrende Autos?*, 2017 ZIS 599; for a moral philosophical point of view see Alexander Hevelke & Julian Nida-Rümelin, *Selbstfahrende Autos und Trolley-Probleme*, 19 Jahrbuch für Wissenschaft und Ethik 5 (2015); see also Sabine Gless & Emily Silverman & Thomas Weigend, *If Robots Cause Harm, Who Is to Blame? Self-Driving Cars and Criminal Liability*, 19 New Criminal Law Review, 412 (2016) with a focus on product liability in the case of negligence.

11 Edmond Awad & Sohan Dsouza & Richard Kim et al., *The Moral Machine experiment*, 563 Nature 59 (2018), who collected 39.61 million decisions with participants from 233 countries, dependencies, or territories.

12 Awad & Dsouza & Kim et al., *ibid*, 59, 61 et seq.; similar results were obtained by Jean-Francois Bonnefon & Azim Shariff & Iyad Rahwan, *The Social Dilemma of Autonomous Vehicles*, 352 (6293) Science 1573, 1574 (2016).

13 Thus, the preference for younger people in the Asian cultural area is less pronounced.

14 E.g. Edmond Awad & Bence Bago & Jean-Francois Bonnefon et al., 7(2) MDM Policy & Practice 1 (2022) with four nationally representative panels (Brazil, France, Japan, and the United States) of 1000 participants; Joaquin Navajas & Facundo Álvarez Heduan & Gerry Garbulsky et al., *Moral responses to the COVID-19 crisis*, 2021 Royal Society Open Science 8:210096 with 15,420 participants from 10 Latin American countries.

15 For example, Austria, Switzerland, Spain, Portugal, Latin American countries such as Argentina, Brazil and Mexico, Japan, South Korea and Taiwan.

16 *Tatbestandsmäßigkeit* can be translated roughly as *actus reus* plus *mens rea*; cf. Michael Bohlander, *Principles of German Criminal Law*, pp. 16 et seq. (2008).

17 Cf. Albin Eser, *Justification and Excuse*, 24 American Journal of Comparative Law 621, 623 et seq. (1976); Dubber, supra note 1, 680 et seq.

18 For example, George P. Fletcher, *Basic Concepts of Criminal Law* 6.3 (1998); George P. Fletcher, *The Grammar of Criminal Law I*, pp. 51 et seq. (2007); Markus D. Dubber, *An Introduction to the Model Penal Code,* 3.2, p. 25, footnote 72*, *(2^nd^ ed. 2015); see also Russell Christopher, *Tripartite Structures of Criminal Law in Germany and Other Civil Law Jurisdictions*, 28 Cardozo L. Rev. 2675 (2007).

19 Cf. supra notes 6, 8 and Blackstone’s Criminal Practice, 2022, A 3.47 et seq.

20 Albin Eser, supra note 17, 634.

21 RGSt 61, 242.

22 The provisions cover two different scenarios: § 228 BGB is concerned with dangers arising from an object, whereas § 904 BGB deals with damage caused to objects that have no relation to the danger. C.f. Bohlander. supra note 16, p. 107; George P. Fletcher, *Basic Concepts of Criminal Law*, pp. 139 et seq. (1998).

23 Cf. Bohlander*,* supra note 16, p. 129; Ivó Coca Vila, *Conflicting Duties in Criminal Law*, 22 (1) New Criminal Law Review 34, 34 et seq. (2019).

24 RGSt 20, 190. It can already be found in Karl Binding, *Handbuch des Strafrechts, Erster Band*, p. 765 (1885), as a special case of the necessity defence.

25 See Albin Eser, *Justification and Excuse: A Key Issue in the Concept of Crime*, in *Rechtfertigung und Entschuldigung. Rechtsvergleichende Perspektiven, Justification and Excuse, Comparative perspectives*, Vol. I, p. 17, 55 (Albin Eser & George P. Fletcher eds., 1987).

26 The concept of the *Tatbestand* in German law encompasses such diverse issues as the definition of an act in the legal sense, causation, several categories of objective attribution, but also the subjective side of the offence, such as intent, mistake, etc. Cf. Bohlander, supra note 16, p. 29.

27 Bohlander, *Principles of German Criminal Law*, supra note 16, pp. 129 et seq.

28 Josef Kohler*, Das Notrecht*, 8 Archiv für Rechts- und Wirtschaftsphilosophie (ARWP/ARSP), 411, 431 et seq. (1915); rediscovered by Till Zimmermann, *Rettungstötungen*, pp. 192 et seq., footnote 720 (2009). Kohler was opposed to punishment.

29 An even earlier variant can be found in the Habilitation thesis of Karl Engisch, *Untersuchungen über Vorsatz und Fahrlässigkeit im Strafrecht*, p. 288 (1930).

30 Hans Welzel, *Zum Notstandsproblem*, 63 Zeitschrift für die gesamte Strafrechtswissenschaft (ZStW) 48, 51 (1951).

31 Philippa Foot, *The Problem of Abortion and the Doctrine of the Double Effect*, 5 Oxford Review 5 (1967).

32 Judith Jarvis Thomson, *The Trolley Problem*, 94 (6) The Yale Law Journal 1395 (1985).

33 Walter Perron in *Schönke & Schröder, Strafgesetzbuch*, § 34 StGB no. 24 (30th ed. 2019); Volker Erb in *Münchener Kommentar*, § 34 StGB no. 148 (4th ed. 2020) with further details; for Austria: Helmut Fuchs, *Strafrecht AT*, 17th chapter no. 58 (9th ed. 2016); for Switzerland: Günter Stratenwerth & Wolfgang Wohlers in *Schweizerisches StGB*, Art. 17 StGB no. 3 (3rd ed. 2013).

34 Roxin & Greco, supra note 4, § 16 no. 33; see also Ulfrid Neumann, *Necessity/Durres*, in *The Oxford Handbook of Criminal Law*, p. 583, 601 (Dubber & Hörnle, eds., 2014).

35 BVerfGE 115, 118 on § 14 III LuftSiG in the version of Jan. 11, 2005 (BGBl. I, p. 78).

36 For example, Stephan Stübinger, *“Not macht erfinderisch” – Zur Unterscheidungsvielfalt in der Notstandsdogmatik*, 123 ZStW 403 (2011); Perron, supra note 33, § 34 StGB nos. 23 et seq.; see also Tatjana Hörnle, *Hijacked Airplanes: May they Be Shot Down?,* 10 (4) New Criminal Law Review 582, 589 et seq. (2007). To the opposite view in the case of unilateral distribution of the chance of rescue Erb*,* supra note 33, § 34 StGB nos. 152 et seq.; Zimmermann, supra note 28, pp. 386 et seq.; see also Bohlander, supra note 16, pp. 112 et seq.; Ulfrid Neumann, in *The Oxford Handbook of Criminal Law*, supra note 34, p. 602.

37 BVerfGE 115, 118, 130 (supra note 35).

38 See the play by Ferdinand von Schirach, *Terror*, about a fighter pilot who shoots down a Lufthansa airplane to prevent a terrorist from crashing it into the fully occupied Allianz Arena in Munich. The broadcast of the film on TV on October 17, 2016 caused a sensation, but also prompted criticism. In the end, the question of guilt or innocence was put to the audience for a vote; 86.9% of the participants were in favour of acquitting the fighter pilots. The results were similarly clear among audiences in Austria and Switzerland.

39 For example, Joshua Dressler, *Understanding Criminal Law*, § 22.05 (7th ed. 2015); Wayne R. LaFave, *Substantive Criminal Law,* § 10.1(c) (2nd ed. 2003). In § 3.02.1a MPC it simply says: “Conduct that the actor believes to be necessary to avoid a harm or evil to himself or to another is justifiable, provided that: the harm or evil sought to be avoided by such conduct is greater than that sought to be prevented by the law defining the offense charged [...].”

40 Supra notes 6 and 8.

41 For Japan, for example, Akihiro Onagi, *Die Notstandsregelungen im japanischen und deutschen Strafrecht im Vergleich,* pp. 20 et seq. (1993).

42 Walter Gropp, *Strafrecht AT*, § 5 no. 308 (4th ed. 2015); see also Kohler, supra note 28, 432.

43 Cf. for instance Detlev Sternberg-Lieben in *Schönke & Schröder*, supra note 33, Vor § 32 StGB nos. 115 et seq.; Christian Jäger, *Die Abwägbarkeit menschlichen Lebens im Spannungsfeld von Strafrechtsdogmatik und Rechtsphilosophie*, 115 ZStW 765, 778 et seq. (2003); in the English language Bohlander, supra note 16, pp. 129 et seq.

44 See Hans Welzel, *Anm. zu OGHSt 1, 321,* 1949 MDR 373, 375; Eb. Schmidt, *Anm. zu OGHSt 1, 321*, 1949 SJZ 560, 568 et seq.

45 See also Bernd Schünemann, *Kritische Anmerkungen zum tragischen Dilemma im Strafrecht*, 2020 GA 1, 6 et seq.

46 OGH for the British Zone, Urt. v. 5.3.1949 - StS 19/49, OGHSt 1, 321, 331 et seq.; OGH for the British Zone of 23.7.1949 - StS 161/49, OGHSt 2, 117, 120 et seq.

47 See also BGH Urt. v. 28.11.1952 - 4 StR 23/50, 1953 NJW 513.

48 BVerfGE 115, 118, 130 (supra note 35).

49 For the “self-defence-test” as a common figure of reasoning see Albin Eser, supra note 25, p. 32.

50 Thomson, supra note 32, 1396.

51 According to German criminal law, science justifying necessity is not predominately based on the utilitarian view, but on the concept of solidarity, cf. Neumann, supra note 34, p. 589.

52 Cf. footnote 9.

53 Dtsch. Ethikrat (Ed.), *Solidarity and Responsibility during the Corona Crisis, ad hoc recommendation (Mar. 27, 2020)*; in the English language under: https://www.ethikrat.org/fileadmin/Publikationen/Ad-hoc-Empfehlungen/englisch/recommendation-coronavirus-crisis.pdf. The German Ethics Council is a committee of experts, of whose members half are proposed by the Federal Government and half by the Bundestag, appointed by the President of the Bundestag for four years.

54 *Empfehlungen der Deutschen Interdisziplinären Vereinigung für Intensiv- und Notfallmedizin (DIVI) und sie-ben weiterer Fachgesellschaften* (Recommendations of the German Interdisciplinary Association for Intensive Care and Emergency Medicine and seven other professional societies), second revised version dated Apr. 17, 2020.

55 DIVI *(eds.),* supra note 54, pp. 5 et seq.

56 See for Italy SIAARTI (ed.), *Raccomandazioni di etica clinica per l’ammissione a trattamenti intensivi e per la loro sospensione, in condizioni eccezionali di squilibrio tra necessità e risorse disponibili*, no. 3 (Mar. 23, 2020); for Switzerland SAMW (ed.), *Covid-19-Pandemie: Triage von intensivmedizinischen Behandlungen bei Ressourcenknappheit*, p. 5 (2nd updated version of Mar. 24, 2020), including oncological disease with a life expectancy of less than 12 months and age over 85 years as exclusion criteria.

57 DIVI (eds.), supra note 54, pp. 3, 6 et seq.

58 This is pointed out by Engländer & Zimmermann, supra note 9, 1398; Sternberg-Lieben, supra note 9, 629.

59 German Constituional Court of 16 December 2021, 1 BvR 1541/20.

60 Cf. Bundestag-Drucksache 20/3877.

61 Gutmann & Fateh-Moghadam, supra note 9, 131.

62 Rönnau & Wegner, supra note 9, 404; Sternberg-Lieben, supra note 9, 630 rightly refer to this.

63 However, note Jescheck & Weigend, supra note 7, § 33 V 1 c and V 2; Hans-Ullrich Paeffgen & Benno Zabel in *Nomos-Kommentar zum StGB*, Vor § 32 StGB, nos. 174, 295 (5th ed. 2017); Thomas Fischer, *StGB*, Vor § 32 StGB no. 11a (70th ed. 2023).

64 Cf. Coca Vila, supra note 23, p. 34; to the “self-defence-test” as a figure of reasoning, see Eser, supra note 25, p. 32.

65 Frankfurter Allgemeine Zeitung (FAZ) of Nov. 24, 2019 (“Violence in emergency rooms – when the patient attacks the doctor”).

66 Gaede et al., supra note 9, 133 et seq.; Jäger & Gründel, supra note 9, 153.

67 Roxin & Greco, supra note 4, § 16 no. 122; Kristian Kühl, *Strafrecht AT*, § 18 no. 137 (7th ed. 2012); Ulfrid Neumann, *Der Rechtfertigungsgrund der Kollision von Rettungsinteressen,* in *Festschrift für Claus Roxin zum 70. Geburtstag am 15. Mai 2001*, pp. 421, 430 et seq. (Schünemann ed., 2001); Ulfrid Neumann in *Nomos-Kommentar zum StGB*, supra note 63, § 34 StGB no. 133; Walter Gropp, *Die Pflichtenkollision: weder eine Kollision von Pflichten noch Pflichten in Kollision*, in *Festschrift für Hans Joachim Hirsch zum 70. Geburtstag*, pp. 207, 215 et seq. (Weigend Ed., 1999); Harro Otto, *Die strafrechtliche Beurteilung der Kollision rechtlich gleichrangiger Interessen*, 2005 Juristische Ausbildung (Jura) 470, 472.

68 Georg Freund, *Strafrecht AT*, § 6 nos. 95 et seq. (2nd ed. 2008); Andreas Hoyer, *Strafrechtsdogmatik nach Armin Kaufmann*, p. 145 (1997); Jan C. Joerden, *Dyadische Fallsysteme im Strafrecht*, pp. 82 et seq. (1986); Schuster, supra note 10, 2019 DAR 6, 11. Jäger & Gründel, supra note 9, 160 express sympathy for this solution.

69 *Digest of Justinian* 50.17.185.

70 In this direction also Dtsch. Ethikrat (ed.), supra note 53, p. 4.

71 Engländer & Zimmermann, supra note 9, 1402; Jäger & Gründel, supra note 9, 161.

72 For a legal regulation, see Gaede et al., supra note 9, 131.

73 On the ethical justifiability of this criterion see Hoven, supra note 9, 451; critical. Taupitz, supra note 9, 448.

74 Partially acknowledged by Taupitz, supra note 9, 449.

75 Cf. Tatjana Hörnle, *Priorisierung von Geimpften?*, Ethik Med. 2022 Aug 9.

76 Critical to this criterion is Sternberg-Lieben, supra note 9, 633 et seq.

77 Taupitz, supra note 9, 449; Till Zimmermann, Legal Tribune Online Mar. 23, 2020 *(“Ärzte in Zeiten von Corona – Wer stirbt zuerst?”*).

78 Engländer, *Die Pflichtenkollision bei der Ex-ante-Triage*, in Triage in der Pandemie (supra note 9), pp. 111, 139 et seq.; Rönnau & Wegner, supra note 9, 405.

79 Gutmann & Fateh-Moghadam, supra note 9, p. 132.

80 Cf. Thomas Rönnau in *Leipziger Kommentar zum StGB*, Vor § 32 StGB no. 117 (13th ed. 2019). This applies regardless of whether the conflict of duties excludes the objective side of the offense or is considered justifying, except for those representatives in the literature (cf. footnote 63) who assume this is simply an excuse.

81 Engländer & Zimmermann, supra note 9, 1400; see also BGHSt 62, 223 regarding the guidelines for the distribution of donor organs.

82 Engländer & Zimmermann, supra note 9, 1402; Rönnau & Wegner, supra note 9, 405; Jäger & Gründel, supra note 9, 153 et seq.

83 Cf. Bundestag-Drucksache 20/3877, p. 20.

84 See also Jäger & Gründel, supra note 9, 153; Merkel & Augsberg, supra note 9*,* 708 et seq.; in a general context Coca Vila, supra note 23, 64.

85 DIVI (ed.), supra note 54, p. 4.

86 SAMW (ed.), supra note 56, p. 6.

87 British Medical Association, *COVID-19 – ethical issues. A guidance note*, p. 3, 6 (Apr. 1, 2020*)*.

88 Dtsch. Ethikrat (ed.), supra note 53, p. 5.

89 Cf. Bundestag-Drucksache 20/3877, p. 23.

90 Engländer & Zimmermann, supra note 9, 1401.

91 Sowada, supra note 9, 457; Jäger & Gründel, supra note 9, 157; see also Gaede et al., supra note 9, 135; Hoven, supra note 9, 453 et seq. and Hörnle, *Ex-post-Triage: Strafbar als Tötungsdelikt?*, in Triage in der Pandemie (supra note 9), pp. 149, 166 et seq.

92 Claus Roxin, *Strafrecht Allgemeiner Teil II*, § 31 no. 116 (2003).

93 Also critical in a general context is Coca Vila, supra note 23, 45.

94 Sowada, supra note 9, 458; Mitsch, supra note 9, 326. For the opposite view see Hörnle, *Ex-post-Triage: Strafbar als Tötungsdelikt?*, in Triage in der Pandemie (supra note 9), pp. 149, 166 et seq., who emphasises that the patient is receiving an ongoing medical care with high personnel expenditure.

95 Sternberg-Lieben, supra note 9, 636.

96 Sowada, supra note 9, 459.

97 Engländer & Zimmermann, supra note 9, 1402; Engländer, *Die Pflichtenkollision bei der Ex-ante-Triage*, in Triage in der Pandemie (supra note 9), pp. 111, 138 et seq,; Hörnle, *Ex-post-Triage: Strafbar als Tötungsdelikt?*, ﻿﻿﻿﻿﻿﻿ibid﻿﻿﻿, pp. 149, 183 et seq.; Heinrich, *Die strafrechtliche Relevanz sachfremder Erwägungen für die Triage-Entscheidung*, in Triage in der (Strafrechts-)Wissenschaft (supra note 9), pp. 149 et seq. For the opposite view see Weigend *Auf dem Weg zu einer Regelung der Triage*, *ibid*, p. 375, 394.

98 Cf. Arzt, *Ignorance or Mistake of Law*, 24 (4) The American Journal of Comparative Law 646–679 (Autumn 1976); Bohlander, supra note 16, pp. 119 et seq.

99 For the strict conditions, see Frank Peter Schuster in *Schönke & Schröder*, supra note 33, § 17 StGB nos. 13 et seq.

100 DIVI (ed.), supra note 54, p. 8.

101 For a general context see also Coca Vila, supra note 23, 52 (principle of individualizing misfortune).

102 Engländer, supra note 10, 609.

103 Engländer, supra note 10, 612 et seq.; Volker Erb, *Automatisierte Notstandshandlungen*, in *Festschrift für Ulfrid Neumann zum 70. Geburtstag*, p. 785, 794 (2017); Joerden, in *Autonome Systeme und neue Mobilität,* supra note 10, pp. 73, 82 et seq.; Günther Sander & Jörg Hollering, *Strafrechtliche Verantwortlichkeit im Zusammenhang mit automatisiertem Fahren*, 2017 NStZ 193, 202 et seq.

104 Joerden, supra note 10, pp. 73, 87. However, Engländer considers an excuse to be possible; see supra note 10, 615 et seq.

105 For a general description of the legal framework as a major influencing factor on competition among the largest automobile nations, see also Fraunhofer IAO (Ed.), *Hochautomatisiertes Fahren auf Autobahnen - Industriepolitische Schlussfolgerungen, Studie im Auftrag des Bundesministeriums für Wirtschaft und Energie (BMWi),* pp. 224 et seq. (Oct. 16, 2015).

106 Thomas Burri, *Machine Learning and the Law: 5 Theses* (January 3, 2017), paper accepted at NIPS 2016 (Barcelona); see also Weigend, supra note 10, 603.

107 As a teenager, the *author* used the RANDOMIZE TIMER command in the BASIC programming language to generate always different number sequences with the RND function. Thus, the computer sets a system time dependent quasi-random start value (depending on the first user action). Modern programming languages can be handled in a similar way.

108 Weigend, supra note 10, 602.

109 Schuster, supra note 10, 2017 RAW 13, 15.

110 Awad & Dsouza & Kim et al., supra note 11, 61 et seq.; similar results were obtained by Bonnefon & Shariff & Rahwan, supra note 12, 1574. See also note 38.

111 For a result similar to this one (but with different dogmatic construction) see Gless & Janal, supra note 10, 575; Hörnle & Wohlers, supra note 10, 24 et seq.; Ulfrid Neumann*, Die Programmierung autonomer Fahrzeuge für Dilemmasituationen – ein Notstandsproblem?*, in *Zehn Jahre ZIS*, pp. 393, 404 et seq. (Rotsch Ed., 2018); Weigend, supra note 10, 603 et seq. (justifying collision of duties); from a moral–philosophical point of view Hevelke & Nida-Rümelin, supra note 10, 5. Furthermore, see Eric Hilgendorf, *Autonomes Fahren im Dilemma. Überlegungen zur moralischen und rechtlichen Behandlung von selbsttätigen Kollisionsvermeidesystemen*, in *Autonome Systeme und neue Mobilität,* supra note 10, pp. 143, 164 et seq. (permitted risk, but different from the situation assessed here to be negligence); in this direction see also Susanne Beck, *Das Dilemma-Problem und die Fahrlässigkeitsdogmatik*, *ibid*, p. 117.

112 Hevelke & Nida-Rümelin, supra note 10, 10 et seq.; cf. also Wolfgang Mitsch*, Die Probleme der Kollisionsfälle beim autonomen Fahren*, 2018 Kriminalpolitische Zeitschrift (KriPoZ) 70, 71, who doubts that § 34 StGB (justifying necessity) is the central norm for evaluation.

113 Hörnle & Wohlers, supra note 10, 23; Schuster, supra note 10, in *Hilgendorf (Ed.)*, pp. 99, 111 et seq.; Schuster, supra note 10, 2019 DAR 6, 11; Weigend, supra note 10, 603; Christoph Wolf, *Von Christian Wolffs ‘Philosophia Practica Universalis’ zur Programmierung von Notstandsalgorithmen*, 132 ZStW 283, 301 (2020).

114 Di Fabio et al*., Bericht der Ethikkommission “Automatisiertes und Vernetztes Fahren”*, pp. 10 et seq. (ethical rules 8 and 9).

115 See Hevelke & Nida-Rümelin, supra note 10, 18.

116 Cf. Hevelke & Nida-Rümelin, *ibid*, 19 et seq.

117 This is the direction Daimler manager *Christoph von Hugo* took in an interview at the Paris Motor Show with the US Internet magazine *Car and Driver* (on the Internet at: http://blog.caranddriver.com/self-driving-mercedes-will-prioritize-occupant-safety-over-pedestrians/). See also Hilgendorf, *Recht und autonome Maschinen – ein Problemaufriss*, in *Das Recht vor den Herausforderungen der modernen Technik* (Hilgendorf & Hötitzsch Eds., 2015), pp. 11, 26 et seq.; Gless & Janal, supra note 10, 574 et seq.

118 See Bonnefon & Shariff & Rahwan, supra note 12, 1575.

119 Cf. Hilgendorf, *Autonomes Fahren im Dilemma. Überlegungen zur moralischen und rechtlichen Behandlung von selbsttätigen Kollisionsvermeidesystemen*, in *Autonome Systeme und neue Mobilität,* supra note 10, pp. 143, footnote 1.

120 Gropp, supra note 42, § 5 nos. 311 et seq.; Gropp, *FS-Hirsch*, supra note 67, pp. 207, 217 et seq.; Günther Jakobs, *Strafrecht AT*, § 15 no. 15a (2nd ed. 1993); Zimmermann, supra note 28, pp. 187 et seq.

121 Philosophia practica universalis: methodo scientifica pertracta, Pars Prior, Halae Magdeburgicae 1744, § 212.

122 Theodor Lenckner, *Der Grundsatz der Güterabwägung als Grundlage der Rechtfertigung*, 1985 GA 295, 304 et seq.; Sternberg-Lieben in *Schönke & Schröder*, supra note 33, Vor §§ 32 ff. StGB no. 76; Hruschka, 1984 JZ 240, 242 et seq.; Neumann, in *Festschrift-Roxin*, supra note 67*,* pp. 421, 429 et seq.; Rudolf Rengier in *Karlsruher Kommentar*, Vor §§ 15, 16 OWiG no. 7 (5th ed. 2018); see also Wolf, supra note 113, 301 et seq. For a detailed statement in the English language, see Bohlander, supra note 16, p. 96; Coca Vila, supra note 23, 42 et seq.

123 Cf. Neumann, supra note 67, pp. 421, 430.

124 Neumann in *Nomos-Kommentar zum StGB*, supra note 63, § 34 StGB no. 133.

125 Statistisches Bundesamt (Ed.), *Fachserie 8 Reihe 7**, Verkehrsunfälle 2021*, p. 50 (2022).

126 Cf. for example Duttge, in *Münchener Kommentar*, supra note 33, § 15 StGB no. 135; Sternberg-Lieben & Schuster in *Schönke & Schröder*, supra note 33, § 15 StGB nos. 144 et seq.; see also Carl-Friedrich Stuckenberg, *Causation*, in *The Oxford Handbook of Criminal Law*, supra note 34, p. 468, 488.

127 Bohlander, supra note 16, pp. 63 et seq.; Greg Taylor, *Concepts of Intention in German Criminal Law*, 24 (1) Oxford Journal of Legal Studies 99, 108 et seq. (2004); see also Thomas Weigend, *Subjective Elements of Criminal Liability*, in *The Oxford Handbook of Criminal Law*, supra note 34, p. 490, 500 et seq.

128 For the moral philosophical point of view see Hevelke & Nida-Rümelin, supra note 10, 11 et seq.

129 Fears and protests were very great when seat belts were introduced; see Der Spiegel of Dec. 8, 1975 (“Sicherheitsgurte: Furcht vor der Fessel”).

130 It is assumed that wearing a safety belt reduces the risk of fatal injuries by at least 50%.

131 Cf. Hevelke & Nida-Rümelin, supra note 10, 12, who make the comparison of vaccination campaigns. Although these typically protect those affected from the relevant diseases, there is also a risk of being seriously affected by the vaccination itself.

132 Christopher W. Bauman & A. Peter McGraw & Daniel M. Bartels & Caleb Warren, *Revisiting External Validity: Concerns about Trolley Problems and Other Sacrificial Dilemmas in Moral Psychology*, 8/9 Social and Personality Psychology Compass 536–554 (2014).

133 Eser, supra note 25, p. 21.

